# Evaluation of Pedicle Screw Position on Computerized Tomography Using Three-Dimensional Reconstruction Software

**DOI:** 10.3390/medicina60122040

**Published:** 2024-12-11

**Authors:** Jiwon Park, Jin S. Yeom, Yeonho Kim, Yoonjoong Hwang, Namkug Kim, Sang-Min Park

**Affiliations:** 1 Department of Orthopaedic Surgery, Korea University College of Medicine, Korea University Ansan Hospital, Ansan 15355, Republic of Korea; 2 Spine Center and Department of Orthopedic Surgery, Seoul National University College of Medicine, Seoul National University Bundang Hospital, Seongnam 13620, Republic of Korea; 3 Shinsegae Seoul Hospital, Seoul 07305, Republic of Korea; 4 Department of Convergence Medicine, Institute of Biomedical Engineering, University of Ulsan College of Medicine, Asan Medical Center, Seoul 05505, Republic of Korea; 5 Department of Radiology, Research Institute of Radiology, University of Ulsan College of Medicine, Asan Medical Center, Seoul 05505, Republic of Korea

**Keywords:** pedicle screw fixation, screw violation, three-dimensional reconstruction, metal artifact

## Abstract

*Background and Objectives*: Recent advances in intraoperative navigation systems have improved the accuracy of pedicle screw placement in spine surgery. However, many hospitals have limited access to these advanced technologies due to resource constraints. In such settings, postoperative computed tomography (CT) evaluation remains crucial for assessing screw placement and related potential complications. Metal artifacts in CT scans often compromise the diagnostic accuracy. This study aimed to develop and validate three-dimensional (3-D) reconstruction software to enhance screw localization accuracy and facilitate its practical clinical application. *Materials and Methods*: This study included two phases: 3-D software development utilizing specific threshold values of Hounsfield units for titanium screws followed by internal validation. For validation, fifty pedicle screws were inserted into porcine lumbar vertebrae with random violation (superior, inferior, medial, or lateral). Three fellowship-trained surgeons evaluated screw positions using both conventional CT bone window settings and the developed software. Additional clinical validation involving 386 pedicle screws from cervical to lumbar spine was performed by two surgeons. *Results:* The software demonstrated significantly higher specificity (83% vs. 63%) and positive predictive value (96% vs. 91%) compared to conventional CT bone window settings, while maintaining 100% sensitivity and negative predictive value. Interobserver reliability was excellent for both methods (0.961 for bone window vs. 0.990 for software). In clinical validation, the software showed superior intraobserver (0.83 vs. 0.74) and interobserver reliability (0.855 vs. 0.513) compared to picture archiving and communication system (PACS) workstation evaluation. *Conclusions*: The developed software provides improved accuracy and reliability in pedicle screw position evaluation through distinct screw outline visualization and metal artifact reduction. Its equipment-independent nature and cost-effectiveness make it particularly valuable for clinical implementation.

## 1. Introduction

Accurate pedicle screw placement is crucial for a successful surgical outcome in fusion surgery. However, pedicle screw insertion is difficult and often results in pedicle wall violations and potential harm to the neural, vascular, and visceral structures. Recent advances in navigation systems, robotics, and artificial intelligence have significantly improved the accuracy of pedicle screw insertion. However, many hospitals have limited access to these advanced technologies due to resource constraints [1,2,3]. Many facilities still rely on traditional freehand or fluoroscopic guidance techniques. Consequently, postoperative computed tomography (CT) remains crucial for evaluating screw positioning, particularly when patients develop postoperative neurological deterioration.

The effectiveness of postoperative CT evaluation is significantly hindered by metal artifacts arising from the pedicle screws, decreasing diagnostic accuracy and making it challenging to determine whether neurological symptoms are attributable to screw malposition [4,5,6,7,8]. Metal artifacts, including beam hardening, scatter, noise, and photon artifacts, detrimentally impact imaging quality and diagnostic confidence [9]. While the use of bone-window settings has been a prevalent approach for mitigating the presence of metal artifacts, complete elimination remains unattainable, and screws often appear larger than their true dimensions [10,11].

Various metal artifact reduction (MAR) techniques have been developed over the decades, including modifications to acquisition and reconstruction process, projection data alterations, and virtual monochromatic imaging [9,11,12,13,14]. However, these MAR techniques face significant limitations: they are integrated into specific CT systems, costly to implement and maintain, and may introduce new artifacts, particularly with titanium alloys commonly used in pedicle screws. In addition, recently developed deep learning algorithms have shown promise in spinal imaging analysis, though their application in postoperative pedicle screw evaluation remains limited due to the implementation complexity [15,16,17].

Given the limitations of the currently available options, this study presents novel three-dimensional (3-D) reconstruction software for enhanced evaluation of pedicle screw positioning in postoperative CT scans. The proposed software focuses on practical clinical implementation, emphasizing cost-effectiveness and universal compatibility across different CT equipment types. In this study, we evaluated the ability of the developed software to visualize pedicle screw violations in postoperative spine and assessed its potential for implementation in clinical practice.

## 2. Materials and Methods

### 2.1. Software Development for a 3-D Reconstruction of Pedicle Screws

**Figure 1:**
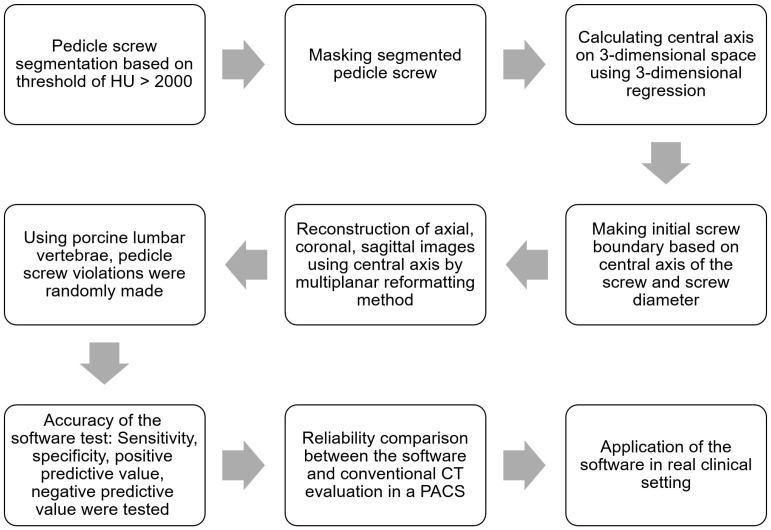
Flow diagram of software development and tests for accuracy and reliability of the software.

The software was developed to overcome metal artifacts in postoperative CT scans for evaluation of pedicle screw position. The development process consisted of four main algorithmic components (Figure 1).

#### 2.1.1. Radiodensity Analysis and Threshold Setting

**Figure 2:**
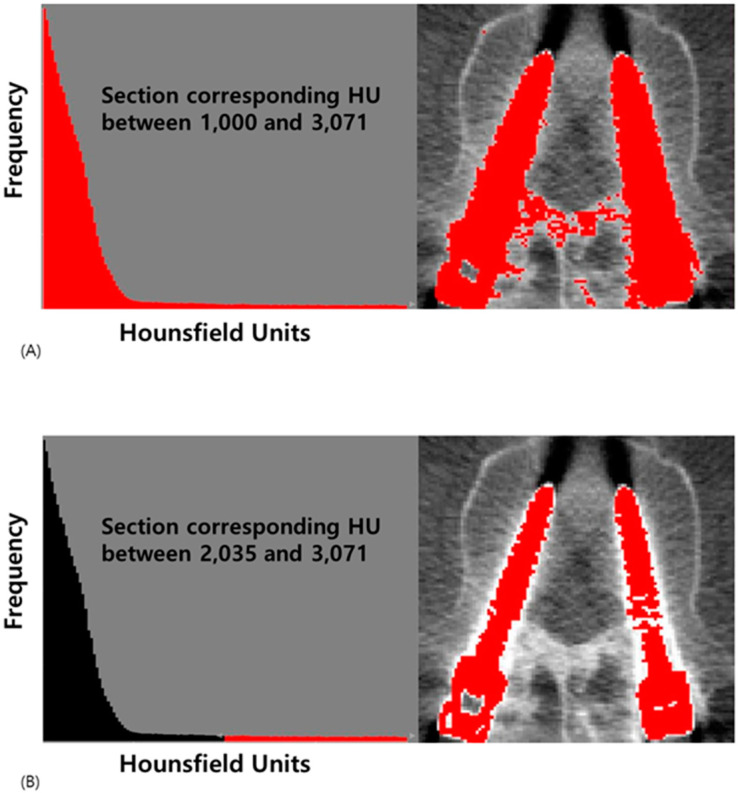
Hounsfield unit histograms of the axial CT images corresponding to the regions of interest. (**A**) The section corresponding to a HU value between 1000 and 3071 is highlighted (**B**) The section corresponding to a HU value between 2035 and 3071 is highlighted.

Initial segmentation utilized histogram analysis of Hounsfield units (HU) to differentiate titanium alloy screws (2300~3071 HU), metal artifacts (1300~3000 HU), and surrounding tissues including cortical bone (1000~2000 HU) [10,11,18]. The ranges of HU for normal substances, metal artifacts, and metal show some overlap but are notably distinct (Figure 2). Based on these distributions, we established a threshold value of 2000 HU for accurate screw material identification.

#### 2.1.2. Screw Head Elimination Process

The reconstruction process began with screw head elimination, where metallic regions were segmented using the established threshold of 2000 HU. The region of interest was manually determined from coordinate intersections, followed by binary region growth from the input point. A sequence of morphological operations including erosion, connected component labeling, and dilation was applied to isolate the screw shaft effectively from the head component.

#### 2.1.3. Central Axis Estimation for Pedicle Screws

**Figure 3:**
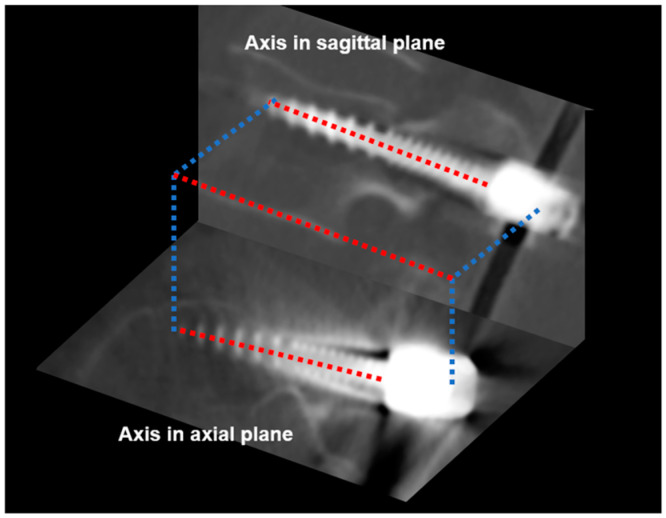
Central axis estimation using least-squares estimation from projection of sagittal and axial planes.

A central axis estimation algorithm was used to estimate a linear equation representing the central axis of the pedicle screws. This was achieved by dividing the segmented screw body and calculating mean coordinates of front and rear sides (approximately 1/4 and 3/4 points). A 3-D straight linear equation was generated using least-squares estimation, with voxels interpolated and projected onto axial and sagittal planes. Least-squares bounding boxes were calculated for the projected positions to ensure accurate axis alignment (Figure 3).

#### 2.1.4. Optimization Voxel Selection for Screw Identification in 3-D Reconstructed Imaging

The algorithm categorizes voxels into three groups: actual screws, metal artifacts, and surrounding tissues, with expected HU values decreasing in that order. Based on this hierarchical distribution, we developed a voxel selection method incorporating screw geometry. The percentage of voxels selected was calculated as

**Figure 4:**
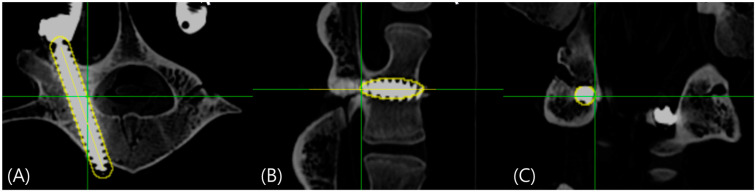
(**A**) Axial, (**B**) sagittal, and (**C**) coronal reconstruction images with screw boundary and central axis using the developed software; threshold > 2000 HU.

**Figure 5:**
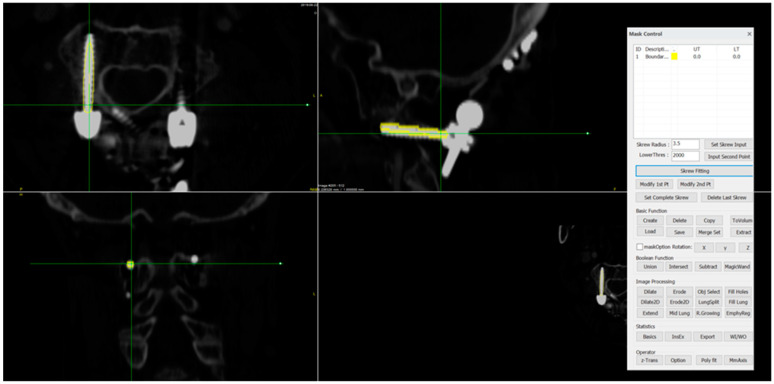
Interface of the developed software.

Selected voxel percentage = (p × p)/[(p + 4)×(p + 4)] × 100 × 0.9,

where p denotes the screw diameter in millimeters. This formula, derived from the geometric ratio of screw volume to surrounding cylindrical space, ensures optimal selection of voxels representing the actual screw structure while minimizing artifact interference (Figure 4 and Figure 5).

### 2.2. Evaluation of Software Reliability and Comparison with Bone Window Setting CT Scans

The reliability of the software was evaluated in two phases: experimental validation using a porcine model and clinical validation using patient data. The experimental phase using a porcine model provided controlled conditions with manual dissection serving as ground truth, enabling accurate assessment of the software’s diagnostic performance through sensitivity, specificity, and predictive values. The clinical phase then validated the software’s reliability in real-world settings, where anatomical verification is not possible but various clinical scenarios exist, including different vertebral levels and screw sizes.

#### 2.2.1. Experimental Phase with Porcine Spine Model

**Figure 6:**
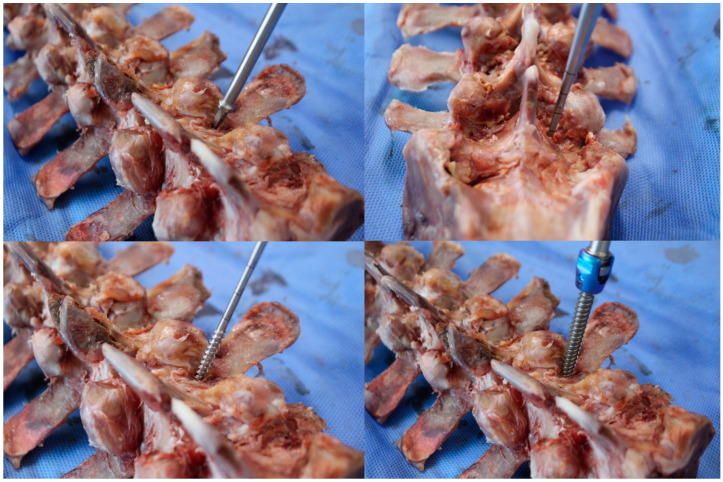
Pedicle screw insertion procedures using freehand technique.

Twenty-five porcine lumbar vertebrae (50 pedicles) were used for experimental validation. Titanium alloy pedicle screws (6.5 mm diameter) were inserted using a freehand technique (Figure 6). The violations were created in a randomized fashion, with the evaluating surgeons blinded to the violation status and location. After completion of the study, manual dissection revealed the actual distribution of violations. For violation assessment, pedicle violations were classified into four grades: Grade 0 indicated no violation with the screw completely contained within the pedicle, Grade 1 represented minor violations (<2 mm) with cortical bulging, Grade 2 indicated moderate violations (2–4 mm) with evident cortical breakage, and Grade 3 represented severe violations with complete pedicle breakage.

Post-insertion CT scans were obtained using a high-resolution protocol (1 mm slice thickness, 0.5 mm intervals). Three fellowship-trained spine surgeons independently evaluated these CT scans using both conventional bone window settings and the developed software, with a two-week interval between assessment methods.

Manual dissection of all specimens served as the reference for determining true violation degree and location. The accuracy of both evaluation methods was assessed using sensitivity, specificity, positive predictive value (PPV), negative predictive value (NPV), and interobserver reliability.

#### 2.2.2. Clinical Evaluation Phase

Unlike the experimental phase where manual dissection provided ground truth, clinical validation focused on evaluating the reliability and consistency of assessment. A retrospective analysis was conducted on postoperative CT scans of patients who underwent pedicle screw fixation. The study included 386 pedicle screws spanning T7-S1 vertebral levels. Two fellowship-trained spine surgeons independently evaluated the screw positions using two methods: (1) conventional CT reconstruction with PACS workstation: observers optimized visualization by adjusting window settings for each case; (2) developed software: observers selected the appropriate screw diameter (5.5 mm, 6.5 mm, or 7.5 mm) based on operative records, then fine-tuned this parameter based on the software-generated screw outline.

To ensure reliable assessment, each observer conducted two separate evaluations for each method, with a minimum four-week interval between readings to minimize recall bias. Reliability was evaluated through (1) intraobserver reliability and (2) interobserver reliability.

The primary outcome measure was the reliability of each evaluation method. Intraobserver reliability was assessed by comparing the two readings from each observer for each method. Interobserver reliability was determined by comparing readings between observers. All reliability measurements were calculated using Cohen’s kappa coefficient with 95% confidence intervals, with statistical significance set at *p* < 0.05.

## 3. Results

### 3.1. Evaluation of Software Reliability and Comparison with Bone Window Setting CT Scans Using Porcine Spine Model

In the porcine model study (50 pedicle screws), violations were intentionally created with the following distribution: 40 screws with violations (31 medial, 4 lateral, 2 superior, and 3 inferior) and 10 screws without violations. Among the violations, 10 were Grade 1 (mild) and 30 were Grade 2 or 3 (moderate to severe).

The software demonstrated superior accuracy compared to conventional CT bone window settings. While both methods achieved 100% sensitivity and NPV, the software showed significantly higher specificity (83% vs. 63%) and PPV (96% vs. 91%). False-positive readings due to metal artifacts were notably reduced with the software (5 vs. 11 out of 120 readings). For medial violations, which are clinically most critical, the software achieved higher specificity (81% vs. 67%) compared to conventional CT (Table 1).

Interobserver reliability was excellent for both methods but slightly superior with the software (kappa = 0.990 vs. 0.961 for conventional CT) (Table 2).

### 3.2. Clinical Evaluation of Software Reliability and Comparison with Bone Window Setting CT Scans

Analysis of 386 screws in 45 patients demonstrated significantly improved reliability with the software compared to conventional PACS workstation evaluation with adjusted window setting. Interobserver reliability was substantially higher with the software (kappa = 0.855, 95% CI: 0.755–0.930) compared to conventional CT (kappa = 0.513, 95% CI: 0.385–0.636). Similarly, mean intraobserver reliability was higher for the software (kappa = 0.838) than conventional CT evaluation (kappa = 0.742) (Table 3).

Both experimental and clinical validations demonstrated that the software provided more consistent and accurate evaluation of pedicle screw positioning while effectively reducing the impact of metal artifacts.

## 4. Discussion

Accurate evaluation of both screw position and the relationship between the screw and adjacent tissues, especially the pedicle cortical wall, is critical to determine whether neurological deterioration is an actual screw-related complication. In addition, screw malposition increases the risk of injury to the vascular and visceral structures owing to the sophisticated structure. While recent advances in navigation systems, robotics, and artificial intelligence have revolutionized pedicle screw placement accuracy in spine surgery, these technologies remain inaccessible for many healthcare facilities worldwide due to high costs and infrastructure requirements [3,19,20,21]. In this context, accurate postoperative evaluation of pedicle screw position using CT imaging remains crucial, particularly in cases of post-surgical neurological deterioration.

This requires the assessment of pedicle screws to be more precise than that of other parts. Metal artifacts pose a significant challenge in evaluating pedicle screw location- in postoperative spinal imaging. To address this issue, several methods for overcoming metal artifacts in CT images have been researched and developed over the past 40 years. For example, several MAR algorithms include metal implant optimization, acquisition improvement, physics-based preprocessing, projection completion, iterative reconstruction, and image postprocessing [2,5,11,14,22,23,24,25,26]. Despite these efforts, some metal objects still pose great challenges for MAR, particularly highly attenuating implants such as pedicle screws. Also, the MAR algorithm may alter the maximum value of HU in the obtained CT data, thereby causing changes in the entire image data. In some cases, applying MAR algorithms to images with severe metal artifacts has resulted in incorrect modifications of non-artifact areas. For titanium alloys, a common material for pedicle screws, the MAR algorithm introduced new and more severe artifacts.

Given these limitation of MAR techniques, most clinicians rely on manual adjustment of bone window setting in the PACS workstation for routine postoperative evaluation of pedicle screw position. This conventional method (typically using a window width (WW) of 2000 HU and a window level (WL) of 500 HU) is widely adopted due to its accessibility and familiarity. However, recent studies have demonstrated that this approach consistently overestimates screw size [27,28]. Research utilizing both in vitro models and clinical cases has shown that titanium alloy screws appear 27~44% larger than the actual size when evaluated using conventional bone window setting [27]. This overestimation can significantly impact the accuracy of violation assessment. Even with ultrawide window settings (WW: 20,000 HU and WL: 3000), screws still appear 9–19% larger than their actual dimensions, demonstrating the inherent limitations of window adjustment techniques [27].

Recent advances in deep learning algorithms have found increasing applications in various medical imaging tasks, including the analysis of radiographs and CT scans for spine imaging analysis [29]. Models based on deep learning, especially convolutional neural networks (CNNs), have been effective in the automated identification and segmentation of spinal structures across different imaging techniques [15,17,25,30,31,32]. These models are commonly utilized for tasks such as recognizing vertebrae and diagnosing conditions such as scoliosis, lumbar spinal stenosis, foraminal stenosis, and spinal tumors. Building on this progress, a novel approach involves the development and validation of a CNN tailored for pedicle screw planning [25,31]. Employing a data-driven strategy, this method analyzes pre-existing planning data from CT-navigated spine instrumentation, aiming to determine screw entry positions by extracting pertinent information from images [25]. The primary goal of this approach is to enhance the accuracy of pedicle screw placement during surgical procedures.

Currently, the application of deep learning algorithms for evaluating postoperative pedicle screw violation remains relatively limited. There was a CNN model, designed for the assessment of intraoperative pedicle screw placement. This model employed the segmentation of screw components, including the head, shaft, and vertebrae [25]. This model demonstrated 93% accuracy for generated radiographs and 83% accuracy for unprocessed radiographs in segmenting pedicle screw shafts [25]. While this study stands as a notable contribution for identifying pedicle screw position, it is considered one of the few endeavors in the realm of evaluating pedicle screw violation using deep learning algorithms. Currently, there is a limited body of research. This might be due to the complexity of development and use. Traditional deep learning models, especially those based on CNNs, often require significant computational resources for both training and inference, and thus it could be challenging to deploy a CNN-based solution for detecting pedicle screw violation.

Given the complexities of MAR algorithms and deep learning-based approaches, this study aimed to address the issue of evaluating pedicle screw positions by developing straightforward 3-D software. Our software demonstrated significant improvements in the accuracy of pedicle screw violation detection compared to conventional CT bone window settings. While both methods achieved 100% sensitivity and NPV, the software showed superior specificity (83% vs. 60–70%) and PPV (exceeding 95% vs. 91%). The software particularly excelled in reducing false positives caused by metal artifacts, which typically make screws appear larger than their actual size in conventional CT imaging. Both methods showed high interobserver reliability in the experimental phase (software: 0.990, conventional CT: 0.961), suggesting consistent evaluation capability.

Of particular clinical significance was the software’s performance in detecting medial violations, which carry the highest risk of neurological complications due to the proximity of the dural sac. The software achieved higher specificity and PPV for medial violations compared to conventional CT, although improvements in detecting inferior violations were limited due to the small sample size. The study utilized high-resolution CT scanning (1 mm thickness, 0.5 mm intervals), which may have contributed to the overall high reliability of both methods.

The software’s clinical validation in 386 screws confirmed its practical utility. In this real-world setting, where screw diameters varied and exact specifications were not always available, the software maintained superior reliability compared to conventional CT evaluation. Interobserver reliability was notably higher with the software (0.855 vs. 0.513), as was intraobserver reliability (0.838 vs. 0.742), demonstrating consistent performance across both controlled experimental and actual clinical conditions.

A key advantage of the developed software lies in its practical implementation and accessibility. This software addressed the limitation of conventional bone window setting by automatically providing optimal visualization of pedicle screws without requiring manual window adjustment. This standardized approach eliminates operator-dependent variability in window level settings and ensures consistent screw visualization across different observers (κ = 0.990 in experimental and κ = 0.855 in clinical settings), enhancing diagnostic reliability. While both the bone window setting method and this software require manual interpretation of screw position, this software provides significant advantages by generating standardized screw outlines based on actual screw dimensions and a fixed HU threshold. This software serves as a standardized platform for interpretation, reducing observer-dependent variations while maintaining the necessary cca judgment in screw position assessment.

In addition, unlike existing MAR algorithms that require specific CT equipment integration or deep learning solutions demanding substantial computational resources, or highly advanced navigation or robotic systems, this software is very simple and cost-effective. It can be integrated into any existing PACS system regardless of CT equipment specifications. Its cost-effective implementation allows widespread adoption, particularly in settings where advanced navigation systems are unavailable. This window-based standalone application can be easily installed on existing PACS workstations without requiring additional hardware or specialized CT equipment, without the need for expensive hardware modifications or system integration. This makes it a highly accessible solution for hospitals of all sizes.

The software does have limitations. The manual selection of reference points for central axis calculation and the predefined threshold values (2000 HU) may introduce some variability in screw boundary determination. Additionally, the current version’s performance in the sagittal plane may be affected by CT slice thickness and intervals.

Despite these limitations, the developed software represents a practical bridge between traditional CT evaluation methods and more advanced technologies, which is very cost-effective. This software can be easily installed, and its DICOM-based implementation enables high accessibility. It can be integrated into existing systems without the need for specialized equipment and can be readily used in clinical settings, making it advantageous in terms of usability. This is particularly relevant in settings where advanced surgical technologies are unavailable or in cases requiring postoperative assessment of procedures performed without navigation assistance.

## 5. Conclusions

This study introduces an innovative approach to overcome the limitations of current imaging techniques, particularly in relation to metal artifacts in CT scans. The development of 3-D reconstruction software tailored for pedicle screws represents a novel solution that leverages the unique radiodensity properties of the titanium alloys used in these screws. The developed software presents a promising solution for enhancing the accuracy of pedicle screw placement assessment, which is crucial for patient outcomes and surgical success. It can be a valuable complementary tool even in the era of advanced navigation.

## Figures and Tables

**Table 1:** Sensitivity, specificity, positive predictive value, and negative predictive value.

		Bone Setting (%)	Developed Software (%)
Observer 1	Sensitivity	100	100
	Specificity	60	90
	PPV	91	98
	NPV	100	100
Observer 2	Sensitivity	100	100
	Specificity	60	90
	PPV	91	95
	NPV	100	100
Observer 3	Sensitivity	100	100
	Specificity	70	80
	PPV	93	95
	NPV	100	100
Overall	Sensitivity	100	100
	Specificity	63	83
	PPV	91	96
	NPV	100	100

PPV indicates positive predictive value; NPV, negative predictive value; sensitivity, specificty, PPV, and NPV were calculated as follows: sensitivity = TP (TP + FN); specificity = TN/(TN + FN); PPV = TP/(TP + FP); NPV = TN/(TN + FN), where TP indicates true positive; TN, true negative; FP, false positive, and FN; false negative.

**Table 2:** Sensitivity, specificity, positive predictive value, and negative predictive value based on pedicle violation location.

Location	Sensitivity (%)		Specificity (%)		PPV (%)		NPV (%)	
	Bone Setting	Developed Software	Bone Setting	Developed Software	Bone Setting	Developed Software	Bone Setting	Developed Software
Superior	100	100	100	100	100	100	100	100
Inferior	100	100	98	100	82	100	100	100
Medial	100	100	67	81	91	100	100	100
Lateral	100	100	100	100	100	100	100	100
Overall	100	100	63	83	91	96	100	100

PPV indicates positive predictive value; NPV, negative predictive value.

**Table 3:** Interobserver and intraobserver reliability of a traditional CT reconstruction method and the developed software for evaluating pedicle screw position.

		Mean Kappa	95% Confidence Interval
Interobserver reliability	CT	0.513	0.385–0.636
	Software	0.855	0.755–0.930
Intraobserver reliability of CT	Observer 1	0.763	0.680–0.846
	Observer 2	0.722	0.591–0.829
	Mean	0.742	
Intraobserver reliability of the software	Observer 1	0.810	0.707–0.892
	Observer 2	0.867	0.762–0.943
	Mean	0.838	

## Data Availability

The data presented in this study are only available on request from the corresponding author due to institutional review board (IRB) restrictions regarding CT imaging data that require additional ethical review prior to sharing.
